# Editorial

**DOI:** 10.1120/jacmp.v11i4.3472

**Published:** 2010-10-13

With the publication of this issue of the *Journal of Applied Clinical Medical Physics*, we conclude the publication of our eleventh volume. In the past several years we have seen a profound growth in the JACMP. In 2008, the last year that my predecessor, Michael Mills, was Editor‐in‐Chief, we received a total of 142 submissions, or an average of 35.5 manuscripts per quarter. This number was exceeded by almost 25% during the first three quarters of this year. We are well on our way to receiving over 200 submissions for this calendar year. Our rejection rate remains at approximately 45%, so we anticipate significant growth in the number of manuscripts published in the JACMP.

We are not anticipating a large number of changes going into next year. The following is one important change we are instituting for manuscripts submitted effective January 1, 2011: if any humans are involved in a study, appropriate approval from the author's institutional review board must be obtained. Authors need to indicate in the manuscript that such approval has been obtained. This approval also includes use of patient data such as treatment plans. This requirement is consistent with editorial policies in most other journals. For details about the policy, prospective authors are advised to review the Submission Guidelines.

In addition, we are trying to limit the size of the JACMP. Rather than increasing our rejection rate, we are encouraging a limit 5000 words for each article. This length restriction will be a recommendation, not a requirement. Associate Editors and reviewers will be reminded of this length restriction when a manuscript is sent out for review. Authors of manuscripts that are significantly longer than 5000 words will be encouraged to restrict the length, especially by reducing the length of the Discussion section.

Finally, when mentioning Associate Editors and reviewers, I would like to acknowledge their difficult and time‐consuming work in maintaining the quality of manuscripts we publish in the JACMP. Names of Associate Editors can be found below, and names of reviewers can be found in a Supplementary File.

Associate Editors (including Guest Associate Editors) for Volume 11 include the following: Nzhde Agazaryan, Salahuddin Ahmad, John Antolak, Sam Beddar, Pat Cadman, Marco Carlone, Nathan Childress, Geoff Clarke, Laurence Court, Luca Cozzi, Larry DeWerd, Bill Erwin, David Followill, Kent Gifford, Michael Gossman, Rebecca Howell, Geoff Ibbott, Jennifer Johnson, Stephen Kry, James Marbach, Mary Martel, Osama Mawlawi, Charles Mayo, Moyed Miften, Eduardo Moros, Firas Mourtada, Ben Nelms, Jennifer O'Daniel, Tinsu Pan, Niko Papanikolaou, Matt Podgorsak, Joann Prisciandaro, Jim Rodgers, John Rong, Yi Rong, Isaac Rosen, Surendra Rustgi, Bill Salter, Mehrdad Sarfaraz, Jeff Shepard, Chengyu Shi, Alf Siochi, Eric Slessinger, Jason Stafford, Frank Van Den Heuvel, Lu Wang, Chuck Willis, Al Zacarias, and Ron Zhu.

Thanks are extended to these individuals and reviewers, and especially to the authors, without whose contributions we would have no journal.

George Starkschall, PhD

Editor‐in‐Chief

November 15, 2010

